# Effect of different surface treatments and multimode adhesive application on the Weibull characteristics, wettability, surface topography and adhesion to CAD/CAM lithium disilicate ceramic

**DOI:** 10.1590/1678-7757-2020-0122

**Published:** 2020-11-27

**Authors:** Karina Barbosa Souza, Dayanne Monielle Duarte Moura, Sarah Emille Gomes da Silva, Gabriela Monteiro de Araújo, Rafael de Almeida Spinelli Pinto, Fabíola Pessôa Pereira Leite, Mutlu Özcan, Rodrigo Othávio de Assunção e Souza

**Affiliations:** 1 Universidade Federal do Rio Grande do Norte Departamento de Odontologia NatalRN Brasil Universidade Federal do Rio Grande do Norte (UFRN), Departamento de Odontologia, Natal / RN, Brasil.; 2 Universidade Federal de Juiz de Fora Departamento de Odontologia Juiz de ForaMG Brasil Universidade Federal de Juiz de Fora (UFJF), Departamento de Odontologia, Juiz de Fora, MG, Brasil.; 3 University of Zurich Division of Dental Biomaterials Clinic for Reconstructive Dentistry, Center of Dental Medicine Zurich Switzerland University of Zurich, Division of Dental Biomaterials, Clinic for Reconstructive Dentistry, Center of Dental Medicine, Zurich, Switzerland.

**Keywords:** Dental Ceramics, Resin Cement, Shear Strength

## Abstract

This paper aims to evaluate the effect of different surface treatments on surface topography, wettability, and shear bond strength of resin cement to glass ceramic.

**Methodology:**

For SBS test, 32 blocks (7x7x2 mm) of lithium disilicate were obtained and randomly divided into eight groups (four blocks per group) according to each surface treatment (HF 20 s, 60 s, 120 s + silanization/S or Scotch Bond Universal/ SBU) and the Monobond Etch & Prime - MEP application followed or not by SBU. On each treated surface ceramic block, up to four dual-curing resin cement cylinders were prepared and light-cured for 40s (N=120/n=15). The specimens were thermocycled (10,000 cycles, 5-55°C, 30 s) and the SBS test (50KgF, 0.5 mm/min) was performed. Furthermore, failure analysis, wettability, AFM, and SEM were carried out. SBS data (MPa) were analyzed using Student's t-test, two-way ANOVA, Tukey's test (5%) and Weibull's analysis.

**Results:**

For HF experimental groups, two-way ANOVA presented the factors “etching time” and “bonding agent” as significant (p<0.05). After silane application, the HF groups presented similar bond strength. SBU application compromised the SBS, except for 120s etching time (HF120sS: 23.39ᵃ±6.48 MPa; HF120sSBU: 18.76ᵃ±8.81MPa). For MEP groups, SBU application did not significantly affect the results (p=0.41). The MEP group presented the highest Weibull modulus (4.08^A^) and they were statistically different exclusively from the HF20sSBU (0.58^B^).

**Conclusion:**

The HF 20s, 60s, 120 s followed by silane, promoted similar resin-bond strength to ceramic and the SBU application after HF or MEP did not increase the SBS.

## Introduction

Lithium disilicate glass ceramics are among the most reliable restorative materials for indirect restorations, both for aesthetic and functional purposes, due to their biocompatibility, favorable appearance, and mechanical properties.^1^^,^^2^ Studies have reported excellent performance for anterior and posterior crowns (100% in a five-year follow-up and 94.8% after eight years),^3^^,^^4^ onlays and inlays (98.9% after five years and 89.6% after 12 years, respectively),^5^ and laminate veneers (82% to 96% after 10 to 21 years).^6^ In this context, Rosetta SM lithium disilicate ceramic (Hass, Gangneung, Korea) has been introduced, which, according to the manufacturer, presents high translucency, opalescence, and resistance due to its microcrystal structure, providing greater performance and simplified fabrication technique using computer-aided design/computer-aided manufacturing (CAD/CAM).^7^

Despite the excellent longevity of glass ceramics, issues such as caries at crown margin, cervical faults, fractures, and restoration dislodgement were reported.^8^ Concerning dislodgement, the bond strength and clinical performance of conventional lithium disilicate-reinforced glass ceramics can be affected by adhesive procedures and surface treatment.^9^ The use of hydrofluoric acid (HF) followed by silanization (S) is the most commonly used surface treatment for the cementation of glass ceramics.^10^^,^^11^^,^^12^ However, the action of HF can promote regions of stress concentration in the ceramic and surface porosity,^7^^,^^10^ which can induce fractures. Moreover, excessive acid etching results in excessive number of compromised and loosely adhered crystals, preventing the resin cement from bonding micromechanically to the ceramic, which decreases the bond strength between the two materials.^10^^,^^13^

Several studies have investigated the effect of different HF concentrations,^2^ etching time, and methods^13^^,^^14^ for surface treatment of ceramics. Among alternative methods, the one-component ceramic primer Monobond Etch & Prime (MEP) (Ivoclar Vivadent, Schaan, Liechtenstein) has been used to replace HF etching followed by silanization. According to the manufacturer, this product contains alcohol, ammonium polyfluoride, and methacrylate silane, that allows etching and silanizing the surface in single step, eliminating HF use, reducing procedure time, and providing a long-lasting bond.^13^ Another method is the use of multimode adhesives, such as the Scotch Bond Universal - SBU (3M ESPE / Irvine, CA, USA), which contains MDP monomer, silane, and adhesive system in a single bottle, which could simplify the adhesion of ceramics to resin cement.^15^^,^^16^

According to El-Damanhoury, et al.^14^ (2018), who compared the effect of surface treatment with Monobond Etch & Primer (MEP) and the application of 4.8% HF followed by silane to feldspathic, lithium dissilicate, and hybrid ceramics, the MEP obtained bond strength results similar to the application of HF followed by Monobond Plus. Tribst, et al.^17^ (2018), compared the effect of 10% HF etching and MEP on resin cement bond strength to feldspathic and lithium disilicate ceramics and found that both surface treatments presented similar bond strength. Yoshihara, et al.^18^ (2015) evaluated the efficacy and stability of silane coupling, using it alone or experimentally prepared with the adhesive system, such as in SBU, concluding that the use of silane alone should be recommended as the surface treatment for glass ceramics. Other studies, however, state that SBU promotes satisfactory adhesion to resin cements when used without additional surface treatments.^19^

Studies evaluating the effects of acid etching and the use of simplified bonding agents on the new lithium disilicate ceramic (Rosetta SM) are lacking; the effect of MEP on this ceramic is also unknown. Thus, the purpose of this study was to evaluate the effect of different HF etching strategies (HF 20 s, 60 s, and 120 s + silanization or SBU) and the MEP application with and without SBU, on the surface topography, wettability, and shear bond strength of a lithium disilicate glass ceramic to resin cement. The null hypotheses tested were: 1) the etching time with hydrofluoric acid would not affect the surface topography, wettability, and resin-bond strength to disilicate ceramic; 2) SBU is an effective substitute for silane; 3) The application of SBU after MEP does not improve the shear strength of resin cement to ceramic.

## Methodology

**Figure 1:**
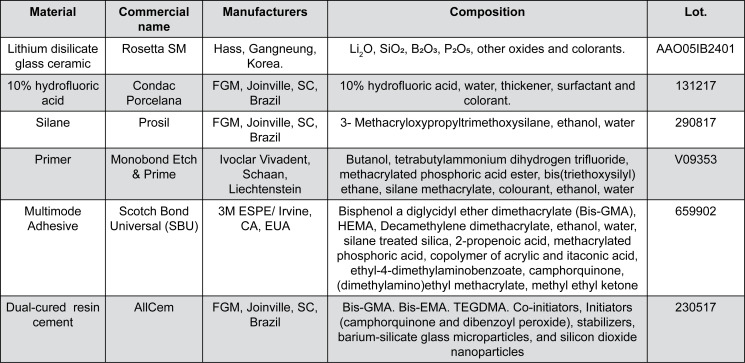
Commercial name, manufacturers, chemical composition, and batch number of materials used in this study

**Figure 2:**
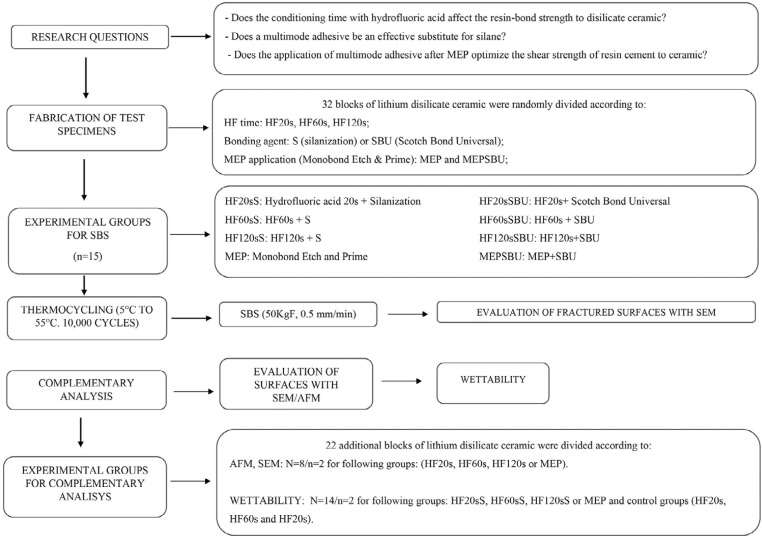
Flowchart of the study protocol. HF: Hydrofluoric acid; S: Silane; SBU: Scotch Bond Universal; MEP: Monobond Etch and Prime; AFM: Atomic Force Microscopy; Scanning Electron Microscopy (SEM); SBS: Shear Bond Strength

The brand, manufacturers, chemical composition, and batch number of the materials used in this study are listed in Figure 1. Figure 2 presents the flowchart of experimental design of this study.

### Ceramic block preparation

Rosetta SM ceramic blocks (15.2x15.2x38 mm) were sectioned using a two-sided diamond disk (Dhpro, Parana, Brazil) in a straight micro-motor (LB100 Beltec, São Paulo, Brazil), under air/water irrigation to obtain 54 smaller blocks, (7x7x2 mm, verified with a digital caliper). The blocks surfaces were regularized with grit SiC abrasive papers (#600, #800 and #1200, 3M ESPE / Irvine, CA, USA) to eliminate disk-related marks during the sectioning. Thereafter, the blocks were ultrasonically cleaned (5 min)—using distilled water—, air-dried and sintered according to the manufacturer's recommendations. In total, 32 blocks were used for the shear bond strength (SBS) test, eight blocks were used for Atomic Force Microscopy (AFM) and scanning electron microscopy (SEM) analysis and 14 blocks for wettability measurements.

### Embedding of samples

The 32 ceramic blocks for SBS were embedded in chemically activated acrylic resin (JET, Dental Articles Classic, Brazil) using a silicone mold (Master-Talmax silicone/Brazil). After resin polymerization, the ceramic blocks surface was polished again with grit SiC abrasive papers (#600, #800 and #1200) in a polishing machine (Labpol 8-12, Extec, USA) until the excess acrylic resin has been removed. Then, the blocks were randomly divided into eight groups (four blocks per group). On each ceramic block, up to four resin cement cylinders were built-up to complete the 15 cylinders per group (n=15). The groups were randomly divided according to “HF time” and “bonding agent”: silanization (HF20sS, HF60sS, HF120sS) or SBU (HF20sSBU; HF60sSBU, Hf120sSBU), and the MEP application (MEP and MEPSBU).

### Surface treatments

Firstly, all specimens were immersed in distilled water and were ultrasonically cleaned for 5 min (Cristófoli Equipamentos de Biossegurança LTDA, Paraná, Brazil). The blocks were left on a gauze to dry for 10 minutes. Then, the adhesive area was delimited by an adhesive tape (Scotch, 3M, Ribeirão Preto, Brazil) with a perforation of 3 mm in diameter. Surface treatments were applied according to the groups (n=15) as follows:

– HF20sS, HF60sS, and HF120sS: The ceramic surface was etched with 10% hydrofluoric acid (Condac Porcelana FGM, Joinville, Santa Catarina, Brazil) during 20 s, 60 s or 120 s, respectively. After, the blocks were washed with air/ water spray for 30 s and dried with jet of air for 30 s (ISO/ TS 11405). Then, a layer of silane agent (Prosil, FGM; Joinville, Santa Catarina, Brazil) was applied with a microbrush (Dentsply, New York, USA) and left for 1 minute followed by jet of air for 30 s to evaporate the solvent according to the manufacturer's recommendations.
– HF20sSBU, HF60sSBU, and HF120sSBU: The ceramic surface was etched with 10% hydrofluoric acid during 20 s, 60 s or 120 s, respectively. After acid etching, a thin layer of multimode adhesive (Scotch Bond Universal/SBU, 3M ESPE/ Irvine, CA, EUA) was applied with a microbrush for 20 s, followed by light jet of air for 5s to evaporate the solvent, and light curing for 40 s (1200 mW/cm^2^ - Radii Cal, SDI, Australia).

MEP: A layer of self-etching ceramic primer (Monobond Etch and Prime, Ivoclar Vivadent, Liechtenstein) was applied to the ceramic surface with a microbrush and rubbed for 20 s. After the action time of 40s, the product was removed with air/water spray for 10s and the surface dried with jet of air. According to the manufacturer's recommendations, it was not necessary to apply silane after MEP.

MEPSBU: The MEP was applied first, followed by the application of the SBU as previously described.

### Resin cement cylinders

For each ceramic surface block, up to four resin cement cylinders (n=15) (Allcem Dual, FGM; Joinville, SC, Brazil) were built on the treated ceramic surface. A Teflon matrix (Ø=2 mm and h=2.0 mm) (Ultradent Jig, Ultradent, South Jordan, UT, USA) was used to standardize the adhesive area and height of the cylinder. After adaptation, the matrix was filled with the resin cement, and light cured from the top of the matrix for 40 s (1200 mW/cm^2^ - Radii Cal, SDI, Australia), and it was chemically cured for 10 min, following the manufacturer's recommendation.^20^ The matrices were removed and the sets (block + resin cement cylinder) were thermocycled.

### Thermocycling and shear bond strength test

All samples were submitted to 10,000 cycles in alternate baths of 5 – 55°C for 30 s each, with a interval of 2 s between immersions (Nova ethics, São Paulo, SP, Brazil, 10.000TC). For the shear bond strength test, the specimen was fixed with a metal device to a universal testing machine (INSTRON 3365, Norwood, USA) so that the resin cement/ceramic interface was perpendicular to the horizontal plane. A chisel-shaped device (Odeme Biotechnology/Brazil) with 50 kg cell was loaded at the resin cement/ceramic interface with a constant speed of 1 mm/min until failure occurred.

The adhesive strength was calculated by the equation: R=F / A, where R=adhesive strength (MPa); F=force (N); A=interfacial area (area of a circle in mm). The adhesive area of each block was defined by the area of a circle, estimated by the following equation: A=πr2, where π=3.14 and r=1 mm.

### Atomic Force Microscopy (AFM) and Scanning Electron Microscopy (SEM)

Eight ceramic blocks were previously prepared as described and they were submitted to the following surface treatments (n=2): HF20s, HF60s, HF120s or MEP. Subsequently, the blocks were submitted to AFM surface analysis (Veeco Multimode, Nanoscope V, Plainview, NY). For AFM, a gold-covered silicon tip (40 μm, 0.01 to 0.025 Ω.cm) was used in the intermittent contact mode. Variations in the vertical position of the tip were recorded as light and dark regions, resulting in superficial topography of the specimens. The tip was maintained in bypass mode at a constant oscillation amplitude (setpoint amplitude). Digital images of 25×25 μm were acquired at a low scanning frequency (1 Hz) for each surface sample.

After AFM, the same samples were examined using a SEM (Hitachi TM 3000, Tokyo, Japan) at 2000x magnification after treatments surfaces.

### Wettability

A total of 14 ceramic blocks (two per group) were previously prepared as described and the were submitted to the following surface treatments: HF20sS, HF60sS, HF120sS or MEP and control groups (HF20s, HF60s and HF120s). The wettability of the ceramic was evaluated by the sessile drop contact angle. Twelve 10-μL drops of distilled water (n=12) were deposited on the ceramic surface (six measurements for each ceramic block) using a dropper adapted to a goniometer. After 5s,^21^ images were taken with a camera (Canon T3i, Canon Lens, Macro 100, Canon, São Paulo, Brazil) coupled at a fixed distance of 30 cm. The mean of contact angle was estimated using a software (Surftens V4.5, OEG, Wildbahn 8i, Frankfurt, Germany).

### Failure mode analysis

The surfaces of the debonded specimens were examined using an optical stereomicroscope 20x (Stereo Discovery V20, Zeiss, Göttingen, Germany) and representative failure modes were analyzed in 50x and 80x SEM (Inspect S50, FEI, Czech Republic). The failure modes were classified as: A) Adhesive in ceramic/resin cement interface; B) Cohesive in ceramic; C) Cohesive in resin cement; D) Mixed 1: adhesive in ceramic/resin cement interface + cohesive in resin cement); E) Mixed 2: adhesive cement/ceramic/ cohesive ceramic.

### Statistical analysis

Statistical assumptions were evaluated prior to the statistical analysis. The power of the sample was estimated with the website www.openepi.com. Data obtained to SBS and wettability were submitted to the statistical model of analysis of variance, after considering the distribution of residues (Levene's test). Shapiro-Wilk test was also performed to evaluate the normality.

For SBS, HF experimental groups were analyzed with two-way ANOVA followed by Tukey's test (5%). Wettability data were analyzed using one-way ANOVA followed by Tukey's test (5%). The MEP groups were compared using Student's *t*-test (5%). All comparisons were carried out using the MINITAB software (Minitab, version 17, 2013, State College, PA, USA). Failure mode, SEM, and AFM underwent descriptive analysis.

The Weibull analysis was performed to evaluate the bond strength reliability, using the Weibull parameter (*m*), characteristic strength (σ_0_), and 95% confidence interval. The Minitab Software (v.17, 2013, State College, PA, USA) was used.

## Results

Levene's test was performed and no statistically significant difference was found among the standard deviations for SBS (*p*=0.4) and Wettability (*p*=0.08). Shapiro-Wilk test indicated the data of the groups follow a normal distribution (*p*=0.12). The power of sample reported was 99.99%. Some samples failed during thermocycling, and they received a bond strength value of 0 MPa. The groups HF20sS and HF60sSBU presented two pretest failures each and the HF20sSBU had five pretest failures. The other groups did not present pretest failures.

### Shear bond strength (SBS)

For HF groups two-way ANOVA revealed that the “etching time” factor (p=0.0001), “bonding agent” factor (p=0.01), and the interaction of both (p=0.0001) were significant (Table 1). For silane application, the HF60sS group (29.35^A^9.0 MPa) presented SBS statically similar to HF20sS (26.27^A^±8.2 MPa) and HF120sS (23.39^A^6.4 MPa). Moreover, SBU application compromised the SBS, except for 120s etching time (HF120sS: 23.39ᵃ6.48 MPa; HF120sSBU: 18.76ᵃ±8.81MPa) and the groups HF60sSBU (7.88^B^±5.9 MPa) presented significantly lower SBS than HF120sSBU (18.76^A^±8.81MPa) and HF660sSBU (17.37^A^±8.64 MPa) (Table 2).

**Table 1:** Results of two-way ANOVA for the “surface treatment” and “bonding agent” factors according to bond strength

Factor	DF	SQ	QM	F	p
Etching time	2	606.49	303.25	4.59	0.0001*
Bonding agent	1	3062.73	3062.3	46.35	0.01*
Etching time X Bonding agent	2	710.94	355.47	5.38	0.006*
Residual	84	5550.59	66.08		
Total	89	9930.76			

* Statistical significance (p<0.05), DF: degrees of freedom; SQ: Sum of square, MS: Mean square, F: F-statistics.

**Table 2:** Means (± SD) of shear bond strength for the groups (n=15) according to the factors: “HF etching time” and “bonding agent.”

	HF etching time		
Bonding agent	HF20s	HF60s	HF120s
Silane	26.27±8.2^aA^	29.35±9.5^aA^	23.39±6.48^aA^
SBU	7.88±5.9^bB^	17.37±8.64^bA^	18.76±8.81^aA^

Lowercase letters: comparison between the same conditioning time and different modes of SBU application (Scotch Bond Universal).

Uppercase letters: comparison between different times of acid conditioning in the same way of SBU application. Tukey's Test (p<0.05).

For MEP groups, student's t-test revealed the MEPSBU (20.25±8,3) and MEP (18.15±5,3) exhibited SBS no significant difference (p=0.41).

**Figure 3:**
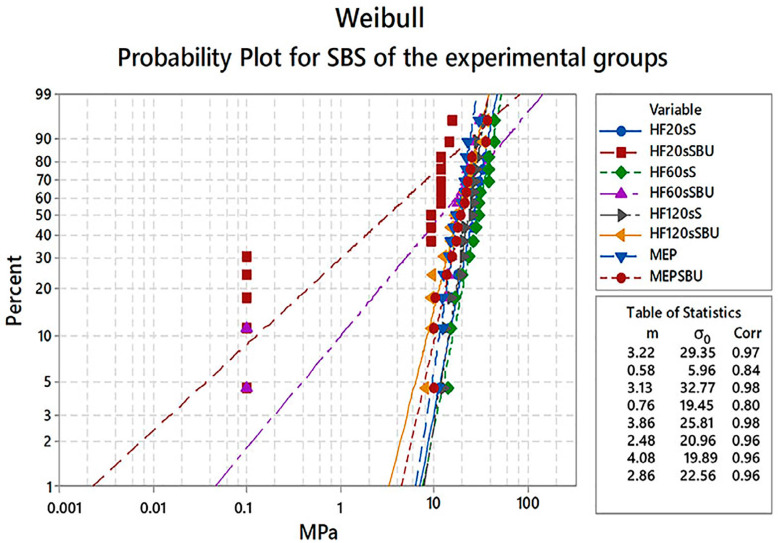
Weibull curves (95% CI) showing the cumulative probability of failures of the different surface treatments tested. m = Weibull modulus, σ0 = characteristic strength, Corr = correction

The Weibull modulus (*m*) and characteristic strength (σ_0_) of all groups were statistically different from each other (p=0.0001). The Weibull distributions are graphically presented in Figure 3 and associated parameters are summarized in Table 3. The MEP group presented the highest Weibull modulus (4.08 MPa)^A^ which was higher than HF20sSBU (0.58 MPa)^B^ but similar to the others. Regarding σ_0_, the HF20sSBU (5.96 MPa)^c^ was lower than HF60sS (32.77 MPa)^ab^ and HF20sS (29.35 MPa)^ab^ groups and similar to the others.

**Table 3:** Characteristic strength (σ_0_), Weibull modulus (m), and 95% CI for shear bond strength according to experimental groups

Group Name	Weibull	95% CI for m	Weibull Characteristic strength (σ0) (MPa)	95% CI for (σ0)
	Modulus (m)			(MPa)
HF20sS	3.22^A^	0.93-5.66	29.35^ab^	24.89 -34.61
HF20sSBU	0.58^B^	0.34-0.98	5.96^c^	2.40 -14.80
HF60sS	3.1^A^	1.96-5.00	32.77^ab^	27.66 -38.83
HF60sSBU	0.76^ab^	0.23-2.50	19.45^abc^	9.39-40.28
HF120sS	3.86^A^	2.42-6.16	25.81^abc^	22.49 -29.63
HF120sSBU	2.48^A^	1.72-3.58	20.96^abc^	16.88-26.02
MEP	4.08^A^	2.90-5.75	19.89^bc^	17.43-22.69
MEPSBU	2.86^A^	2.02-4.06	22.56^abc^	18.69-27.22

Equal uppercase letters indicate statistical similarity among Weibull modulus.

Equal lowercase letters indicate statistical similarity among Weibull characteristic strength (p<0.05).

### AFM and SEM

**Figure 4:**
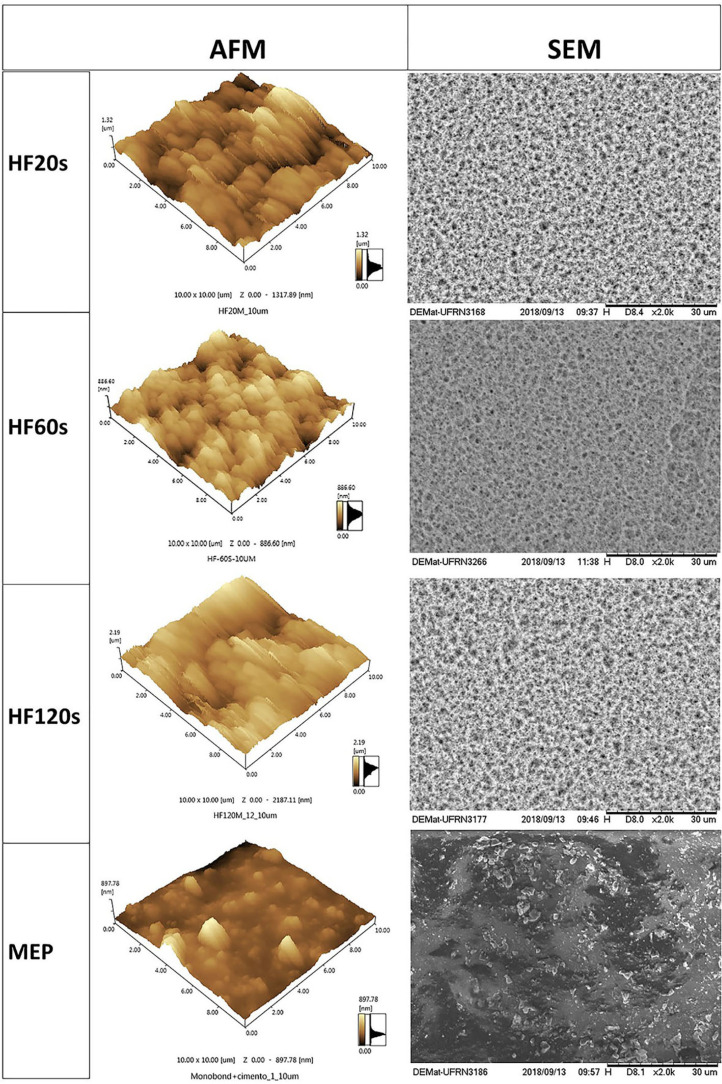
Atomic force microscopy and Scanning electron microscopy images of the HF20s, HF60s, HF120s, and MEP

The AFM images presented irregular surfaces with peaks (lighter areas) and valleys (darker areas) for the 20 s, 60 s, and 120 s groups. For the MEP group, a more uniform surface was observed, with few valleys and peaks (Figure 4).

The SEM etched surfaces for the 20 s, 60 s, and 120 s groups presented irregularities including several microporosities and groves as a result of the dissolution of the glassy phase. However, MEP produced more smooth and homogeneous surface without numerous microporosities as observed in the HF groups (Figure 4).

### Wettability

The one-way ANOVA revealed that the “surface treatment” (*p*=0.0000) was significant. Tukey's test showed that HF20sS (91.56°±11.5)^A^ presented the highest contact angle, being statically similar to the HF60sS (84.25°±3.06)^AB^ and significantly different from the other groups. HF120s (45.75°±11.06)^D^ presented the lowest contact angle and it was statically similar to the HF60s (51.34°±7.06)^CD^. MEP (82.24°±7.63)^B^ presented contact angle values statically similar to both HF60sS and HF120sS (76.10°±2.4)^B^. The results of contact angles are shown in Table 4.

**Table 4:** Means and standard deviation of contact angles (°) in the groups

Group	Bonding Agent	Time	Mean (°)
MEP	-	-	82.24±7.63^B^
HF20s	-	HF 20s	55.18±4.33^C^
HF60s	-	HF 60s	51.34±7.06^CD^
HF120s	-	HF 120s	45.75±11.06^D^
HF20sS	Silane	HF 20s	91.56±11.51^A^
HF60sS	Silane	HF 60s	84.25±3.06^AB^
HF120sS	Silane	HF 120s	76.10±2.40^B^

Equal uppercase letters indicate statistical similarity.

### Failure mode analysis

**Figure 5:**
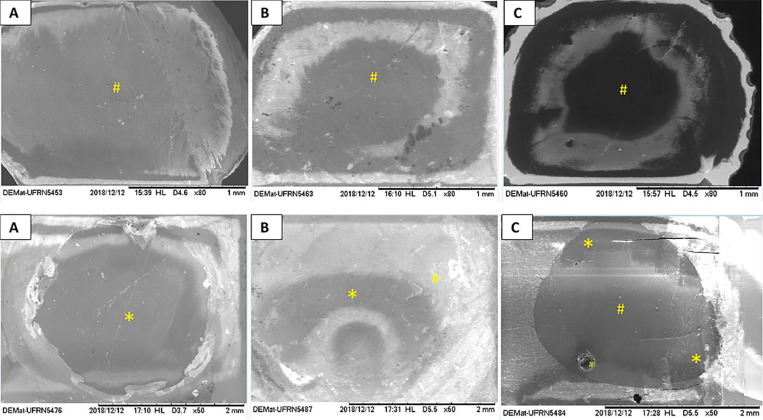
Scanning electron microscopy images (50X and 80x) of failure modes of the ceramics and resin cement cylinders in: A) adhesive at cement/ceramic interface; B and C) adhesive at ceramic/resin cement interface + cohesive at resin cement. *Ceramics; #resin cement

The failure analysis revealed that 92.5% of failures were originated from Mixed 1 mode (predominantly adhesive in resin cement/ceramic interface + cohesive in resin cement) and 7.5% were adhesive in cement/ceramic interface. The percentage of each failure mode for each group tested is shown in Table 5. The representative images are presented in the Figure 5.

**Table 5:** Failure mode analysis and percentage (%) for each experimental group

		Failure Modes		
Groups	Cement ceramic Adhesive	Mixed: adhesive cement/ceramic/ cohesive cement	Pretest failure	Total
HF20sS	3 (20%)	12 (80%)	2	15(100%)
HF20sSBU	-	15 (100%)	5	15(100%)
HF60sS	3 (20%)	12 (80%)	0	15(100%)
HF60sSBU	1 (6.66%)	14 (93.33%)	2	15(100%)
HF120sS	-	15 (100%)	0	15(100%)
HF120sSBU	-	15 (100%)	0	15(100%)
MEP	2 (13.33%)	13 (86.66%)	0	15(100%)
MEPSBU	-	15 (100%)	0	15(100%)

## Discussion

This study objective was to evaluate the effect of different HF etching strategies (HF 20s, 60s, 120s + silanization or SBU) and the MEP with and without application of SBU on the surface topography, wettability, and shear bond strength of resin cement to lithium disilicate glass ceramic. The different surface treatments that precede the cementation of glass ceramic restorations are well discussed in the literature. The application of HF followed by silanization is the most used method for increasing the restoration wettability and improving adhesion to the resin cement.^22^^,^^23^ However, both performance and longevity of the restoration may be adversely affected by acid etching approaches, causing surface defects due to excessive or inefficient etching. Moreover, the adhesion of the resin cement to ceramic can be affected by the bonding agent used.

The shear bond test was chosen to evaluate the bond strength between the ceramic and resin cement. In addition to being cost-effective and easy to implement, it is often used in studies assessing adhesion between two interfaces. In order to reduce non uniform stress distribution in a shear test,^24^ a smaller adhesive area of 2 mm^2^ was used in this study.^25^ All samples in this study were subjected to thermocycling for 10,000 cycles, which simulates conditions equivalent to one year of clinical use.^26^^,^^27^ The fatigue process of thermocycling promotes a faster hydrolytic degradation of the interface due to its contraction and expansion stresses as a consequence of different thermal expansion coefficients among different materials, which is considered a significant predictor of the adhesive performance of restorative interfaces^28^^,^^29^ and for these reasons all groups were submitted to thermocycling.

Based on the results obtained in our study, the first hypothesis that the etching time with hydrofluoric acid does not affect the surface topography, wettability, and resin-bond strength to disilicate ceramic was partially accepted. The SBS results demonstrated that the three HF etching times (20 s, 60 s, and 120 s) did not present significant differences. The HF acts on the ceramic surface increasing the surface energy and the adhesion potential by removing the silica matrix and exposing the structure crystals.^10^^,^^22^^,^^23^ The resulting roughness and increased irregularities, also observed by AFM and SEM results, may favor a micromechanical adhesion by the greater imbrication of resin compounds^30^ added to the chemical adhesion promoted by the bonding agents, increasing overall adhesion. The Weibull analysis also demonstrated that the modulus *m* and σ_0_ of groups with different etching time of 20 s, 60 s, 120 s were similar among them.

According to Puppin-Rontani, et al.^31^ (2017) the exposure time of HF influences the ceramic bond strength. Longer acid etching (40, 60, and 120 s) caused higher dissolution of the glass matrix, creating more irregularities, favoring the micromechanical adhesion of the resin cement to the lithium disilicate crystals. However, some authors report that longer etching times may affect the flexural strength of glass ceramics. Zogheib, et al.^32^ (2011) evaluated the flexural strength of a lithium disilicate ceramic after different acid etching times and found that etching reduced flexural strength and mean values decreased with the increase of HF etching time, which could be related to excessive removal of the glass matrix and consequent ceramic weakening. In this study, although no difference was found in SBS between HF 20 s, 60 s, 120 s times, the 20 s etching time for lithium disilicate ceramics has been recommended by *in vitro*^31^ and clinical studies^33^ for promoting sufficient bonding to cement resin.

Regarding the wettability, our results showed lower contact angle for HF-exclusively groups. The HF etching results in a higher energy surface, due to the removal of contaminants and the increased roughness of the ceramic surface, supported the greater interaction with silane.^10^^,^^22^^,^^31^ In this study, the contact angle for MEP was higher than those in HF etching groups, and similar to the groups with silane (HF60sS and HF120sS), excepting HF20sS, where MEP demonstrated a smaller contact angle. The MEP reduced the surface energy (greater contact angle), which may be an indication that silane molecules contained in the MEP remain effectively linked to the hydroxyl groups available on ceramic surface^34^. According to Moreno, et al.^34^ (2019) MEP produces a more superficial conditioning pattern than HF and this may be responsible for decreasing the ceramic wettability. Some authors report that the presence of fluoride contained in the MEP, also seems to reduce the ceramic wettability,^14^^,^^17^^,^^34^ which may justify our findings. Furthermore, it was observed in this study that longer conditioning times (120 s) produced a smaller contact angle. Ramakrishnaiah and others^10^^,^^23^^,^^30^ demonstrated that wettability is directly proportional to surface irregularities, however, despite the greater degradation of the vitreous matrix by prolonged periods of HF, increasing the exposure of hydroxyl groups^10^^,^^22^ of the ceramic, it can also decrease the interaction of these groups with the silane, and consequently, the hydrophobicity of the ceramic, providing no advantage for adhesion.^17^^,^^34^ However, further studies are necessary to support this statement.

The second hypothesis that SBU is an effective substitute for silane was rejected. According to our results, HF 20 s and 60 s followed SBU were significantly lower than those of other groups. This groups also demonstrated pretest failures during thermocycling which further decreased the SBS. The acid etching procedure, besides increasing micro-retentions, exposes hydroxyl groups that chemically bond to silane coupling agents, improving the overall bond strength to the resin cement.^23^ Moreover, these coupling agents can bond to organic and inorganic materials and also bond to resinous compounds.^35^ On the other hand, multimode adhesives contain silane and MDP monomers for the purpose of simplifying the clinical process.^15^ However, the chemical bonding of their components to ceramic and resin cement without previous treatment with silane can be inefficient, causing lower bond strength values.^36^ It can be attributed to the fact that the silane is hydrolysable and more stable at a pH between 4-5 where the pH of SBU is 2.7. Thus, if a pH solution does not favor the silane stability, its hydrolysis and consequent performance of double functional monomers can be affected, compromising the efficiency of adhesion.^18^^,^^22^^,^^36^

Moro, et al.^35^ (2017) studied the effect of silane application prior to the use of a multimode adhesive on the micro-SBS of a lithium disilicate ceramic and found a significant increase in bond strength compared to the groups treated only with adhesive. The study by Kim, et al.^36^ (2015) evaluated the bond strength of multimode adhesives, including SBU, to a leucite-reinforced glass ceramic. They found that, although the bond strength between ceramic and resin cement was improved with the use of multimode adhesives, the use of silane before the adhesive is preferable, since the chemical interaction of silane contained in the multimode adhesive with ceramic is not as efficient as the silane used by itself. Kalavacharla, et al.^15^ (2015) also evaluated the use of silane prior to the use of a multimode adhesive and its effect on bond strength to a lithium disilicate ceramic, finding similar results to ours and others studies. In our study, Weibull analysis confirmed the results of SBS, where *m* of HF20sSBU group was significantly lower than all experimental groups with HF followed by silane, which can indicate a lower efficiency and reliability of the adhesive interface. Several authors have reported that silane contained in multimode adhesives does not promote an efficient resin-bond strength to ceramic, which corroborates the results of our study. Thus, HF followed by silane remains the most recommended surface treatment for lithium disilicate ceramics.^15^

The third hypothesis, that the application of SBU after MEP would not improve the shear strength of resin cement to ceramic was accepted. For the MEP groups, our results demonstrated non-significant SBS and *m* with and without SBU. This stable adhesion after SBU, probably occurred due to improved chemical retention, since the silane in the MEP and the SBU result in stable adhesion added to the micro-retentions created by the MEP etching. However, considering that the application of SBU after MEP does not improve the SBS, it can be considered a dispensable step. When the MEP was compared to treatments of HF etching surfaces, the results of *m* and σ_0_, demonstrated that the use of MEP provided a similar bond strength. The effect of MEP on the ceramic surface is based on the action of ammonium and silane polyfluoride components, resulting in the creation of micro-retentions added to the silane action on the ceramic, providing a mechanical and chemical bonding of the ceramic with the resin cement, simplifying the procedure with a single product.^37^ Furthermore, MEP has lower toxicity compared to HF, and therefore its use in mouth can be a concern.^38^ Other studies also reported that the MEP can be an alternative for HF followed by silanization, without compromising the bond strength between ceramic and resin cement^14^^,^^17^ providing clinically efficiency and durable adhesion.^39^

Lopes, et al.^40^ (2019) compared different HF concentrations (5, 9.5, 9.6, and 10%) with MEP and their results corroborate with our findings. Prado, et al.^38^ (2018) compared 5% HF etching followed by silanization with MEP and their effects on the bond strength between resin cement and lithium disilicate glass ceramics and feldspathic ceramics and they found that the groups treated with HF obtained the highest means for microshear resistance. The action of the acid present in MEP (ammonium dihydrogen tetrabutyltrifluoride) on the ceramic surface produces less micromechanical retention and irregularities which was also observed in the AFM and SEM results of our study. The acid present in MEP has a milder acidity compared to hydrofluoric acid, which is expected to result in more superficial degradation.^14^

Additional *in vitro* studies varying the pH levels, masticatory load and clinical trials should be performed to verify the longevity of restorations submitted to different surface treatments.

## Conclusion

Based on the results, the following could be concluded:

– HF20s followed by silane is the most suitable surface treatments for lithium disilicate ceramic;
– SBU application after HF or MEP reduced the shear bond strength to lithium disilicate ceramic;
– MEP promoted Weibull modulus and characteristic strength similar to the groups of HF followed by silane.
– The contact angle for MEP was higher than those in HF etching groups but similar to the groups with silane, excepting HF20sS.
